# The preventable burden of mortality from unsafe abortion among female sex workers: a Community Knowledge Approach survey among peer networks in eight countries

**DOI:** 10.1080/26410397.2023.2250618

**Published:** 2023-09-15

**Authors:** Brian Willis, Kathryn Church, Emily Perttu, Heather Thompson, Swarna Weerasinghe, Wendy Macias-Konstantopoulos

**Affiliations:** a Director, Global Health Promise, Portland, OR, USA; b Independent Consultant, London, UK; Honorary Assistant Professor, Department of Epidemiology & Population Health, London School of Hygiene & Tropical Medicine, London, UK.; c Director of Data Analysis, Global Health Promise, Portland, OR, USA; d Maternal Health Advisor, Global Health Promise, Portland, OR, USA; Adjunct Professor, Schulich School of Medicine, McMaster Faculty of Medicine; & Obstetrician/Gynecologist, Grey Bruce Health Services, Hamilton, Ontario, Canada; e Biostatistician, Global Health Promise, Portland, OR, USA; Associate Professor, Department of Community Health and Epidemiology, Dalhousie University, Faculty of Medicine, Halifax, Nova Scotia, Canada; f Global Policy Advisor, Global Health Promise, Portland, OR, USA; Director, Center for Social Justice and Health Equity, Department of Emergency Medicine, Massachusetts General Hospital and Harvard Medical School, Boston, MA, USA

**Keywords:** female sex workers, low- and middle-income countries, unsafe abortion, maternal mortality, sexual and reproductive health services, unintended pregnancy, Africa, Asia, Latin America

## Abstract

Previous studies have found high levels of unintended pregnancy among female sex workers (FSW), but less attention has been paid to their abortion practices and outcomes. This study is the first to investigate abortion-related mortality among FSW across eight countries: Angola, Brazil, Democratic Republic of Congo (DRC), India, Indonesia, Kenya, Nigeria, and South Africa. The Community Knowledge Approach (CKA) was used to survey a convenience sample of FSW (*n* = 1280). Participants reported on the deaths of peer FSW in their social networks during group meetings convened by non-governmental organisations (*n* = 165 groups, conducted across 24 cities in 2019). Details on any peer FSW deaths in the preceding five years were recorded. The circumstances of abortion-related deaths are reported here. Of the 1320 maternal deaths reported, 750 (56.8%) were due to unsafe abortion. The number of abortion-related deaths reported was highest in DRC (304 deaths reported by 270 participants), Kenya (188 deaths reported by 175 participants), and Nigeria (216 deaths reported by 312 participants). Among the abortion-related deaths, mean gestational age was 4.6 months and 75% occurred outside hospital. Unsafe abortion methods varied by country, but consumption of traditional or unknown medicines was most common (37.9% and 29.9%, respectively). The 750 abortion-related deaths led to 1207 children being left motherless. The CKA successfully recorded a stigmatised practice among a marginalised population, identifying very high levels of abortion-related mortality. Urgent action is now needed to deliver comprehensive sexual and reproductive healthcare to this vulnerable population, including contraption, safe abortion, and post-abortion care.

## Résumé

De précédentes études ont révélé des niveaux élevés de grossesse non désirée chez les professionnelles du sexe, mais moins d’attention a été accordée à leurs pratiques d’avortement et aux résultats de celles-ci. Cette étude est la première à enquêter sur la mortalité liée à l’avortement chez les professionnelles du sexe dans huit pays: Afrique du Sud, Angola, Brésil, Inde, Indonésie, Kenya, Nigéria et République démocratique du Congo (RDC). L’approche des connaissances communautaires a été utilisée pour sonder un échantillon de convenance de professionnelles du sexe (*n* = 1280). Les participantes ont signalé le décès d’autres professionnelles du sexe dans leurs réseaux sociaux lors de réunions de groupe constituées par des organisations non gouvernementales (*n* = 165 groupes, dans 24 villes en 2019). Les détails sur les décès d’autres professionnelles du sexe pendant les cinq années précédentes ont été enregistrés. Les circonstances des décès liés à un avortement sont données ici. Sur les 1320 décès maternels rapportés, 750 (56.8%) étaient dus à un avortement à risque. Le nombre de décès signalés liés à un avortement était le plus élevé en RDC (304 décès rapportés par 270 participantes), au Kenya (188 décès signalés par 175 participantes) et au Nigéria (216 décès rapportés par 312 participantes). Pour les décès liés à un avortement, l’âge gestationnel moyen était de 4,6 mois et 75% sont survenus en dehors de l’hôpital. Les méthodes d’avortement à risque variaient selon les pays, mais la consommation de médicaments traditionnels ou inconnus était la plus courante (37.9% et 29.9% respectivement). Les 750 décès liés à un avortement ont fait 1207 orphelins. L’approche des connaissances communautaires est parvenue à enregistrer une pratique stigmatisée au sein d’une population marginalisée, et a identifié des niveaux très élevés de mortalité liée à l’avortement. Des mesures urgentes sont maintenant nécessaires pour prodiguer des soins complets de santé sexuelle et reproductive à cette population vulnérable, y compris en matière de contraception, d’avortement sans risque et de soins post-avortement.

## Resumen

Estudios anteriores han encontrado altos niveles de embarazo no intencional entre trabajadoras sexuales (TS), pero no se ha prestado mucha atención a sus prácticas y resultados de aborto. Este estudio es el primero en investigar la mortalidad relacionada con el aborto entre TS en ocho países: Angola, Brasil, República Democrática del Congo (RDC), India, Indonesia, Kenia, Nigeria y Sudáfrica. Se utilizó el Enfoque de Conocimientos Comunitarios (ECC) para encuestar a una muestra de conveniencia de TS (*n* = 1280). Durante reuniones en grupo convocadas por organizaciones no gubernamentales (*n* = 165 grupos, realizadas en 24 ciudades en 2019), las participantes informaron en las redes sociales sobre las muertes de TS pares. Se anotaron los detalles sobre muertes de TS pares en los cinco años anteriores. Aquí se informa sobre las circunstancias de muertes relacionadas con el aborto. De las 1320 muertes maternas reportadas, 750 (56.8%) fueron atribuidas al aborto inseguro. El número de muertes relacionadas con el aborto reportadas fue más alto en RDC (304 muertes reportadas por 270 participantes), Kenia (188 muertes reportadas por 175 participantes) y Nigeria (216 muertes reportadas por 312 participantes). Entre las muertes relacionadas con el aborto, la edad gestacional media fue de 4.6 meses y el 75% ocurrió fuera del hospital. Los métodos de aborto inseguro variaron por país, pero el consumo de medicamentos tradicionales o desconocidos fueron los más comunes (37.9% y 29.9% respectivamente). Las 750 muertes relacionadas con el aborto tuvieron como consecuencia que 1207 niños perdieran a su madre. El ECC logró registrar una práctica estigmatizada entre una población marginada, e identificó niveles muy altos de mortalidad relacionada con el aborto. Ahora es necesario tomar acción urgente para proporcionar servicios integrales de salud sexual y reproductiva a esta población vulnerable, que incluyen anticoncepción, aborto seguro y atención postaborto.

## Introduction

Unsafe abortion remains a major public health concern, particularly in countries where abortion services remain legally restricted. Just under half of the 55.7 million abortions that occurred annually between 2010 and 2014 were categorised as unsafe by the World Health Organization (WHO), either conducted with an unsafe provider, an unsafe method, or both.^1^ Despite the development and distribution of medical abortifacients (mifepristone and misoprostol), unsafe abortion remains a significant cause of preventable maternal mortality: in its most recent global analysis, WHO estimated that unsafe abortion accounted for 7.9% of all maternal deaths worldwide, and this was higher still in the world’s regions which retain more legal barriers to safe abortion care (9.9% in sub-Saharan Africa and 9.6% in Latin America and the Caribbean).^2^ There also remains a substantive burden of morbidity caused by unsafe abortion, including haemorrhage and sepsis.^1^

Access to safe abortion care is dependent on both the legal and health system context within which abortion is provided, as well as the social and economic context surrounding the unintended pregnancy. From a legal and policy perspective, governments can impose restrictions that disrupt or limit women’s timely access to care, including gestational limits, circumstantial restrictions (e.g. to save a woman’s life or in cases of rape), or regulatory requirements (e.g. requiring multiple physician signatures).^3^ These restrictions can be overcome by those with resources to travel or find safe services, but they are often insurmountable for the socially and economically marginalised. Studies show that women from poorer, more socially marginalised groups are more likely to resort to unsafe methods of abortion than other women.^4^

Female sex workers (FSW) are a population who suffer disproportionately in their access to healthcare, including reproductive healthcare.^5^ This group is poor, stigmatised, and subject to widespread human rights violations.^3^ Consequently, they experience poorer health outcomes than the general population.^6^ They can be discriminated against by health workers and other officials, and may be prevented from accessing care due to illegal migrancy status.^7–9^ As with other marginalised communities, FSW may lack knowledge on their legal entitlements, may be unaware of where safe services are available, or may feel too stigmatised to request abortions from regulated services (if they are available).^10,11^ Social networks have also been found to be instrumental in knowledge dissemination on unsafe abortion practices, and these may be particularly pronounced among marginalised FSW networks.^10,12^ In the great majority of countries, there also remain financial barriers to accessing abortion services, and even the costs of emergency post-abortion care (PAC) can be unaffordable.^6^

FSW are at particular risk of poor sexual and reproductive health (SRH) outcomes due to behavioural and structural factors related to their work, including multiple sexual partnerships, violence, limited ability to negotiate condom use, and limited access to healthcare.^13^ Much of the existing literature on the health of FSW relates to their sexual health, including as both victims and drivers of HIV epidemics. As a key population at risk of contracting and transmitting HIV, SRH programmes have often focused on condom promotion and use in this group.^14^ A 2014 systematic review identifying 54 sex worker programmes in sub-Saharan Africa found that none provided abortion care, and few offered contraception (other than condoms).^15^

Studies point to high levels of unmet need for contraception among FSW, in particular for non-barrier contraception.^5,16^ Studies across a range of contexts suggest unintended pregnancy and abortion are very common in this group, and higher than the national average in most settings, including studies in Brazil,^17^ India,^18,19^ Kenya,^11,16,20,21^ and South Africa.^16^ Studies on abortion experiences and outcomes among FSW in low-and middle-income countries (LMICs) are more limited. Only one study from Cambodia has previously quantified deaths of FSW from abortion, finding that abortions comprised 40% of the reported maternal deaths.^22^ Studies from settings where abortion is legally restricted (Brazil, Tanzania) and more liberal policy contexts (South Africa, Bangladesh) demonstrate frequent informal sector abortion access among FSW, including use of traditional methods/herbs and self-administration with either legal or black-market medical abortion (MA) products.^10,17,23,24^ Studies looking at other abortion outcomes in FSW are very limited: in Brazil, use of unsafe methods resulted in infection and haemorrhagic complications;^17^ in a South African study of informal sector abortion users (a third of whom were FSW), one-third required treatment for complications.^10^ Other studies have also pointed to high rates of complication among FSW following abortion (Bangladesh, 58%).^24^

This current report is an output of a larger study investigating the mortality of FSW in eight LMICs: Angola, Brazil, Democratic Republic of Congo (DRC), India, Indonesia, Kenya, Nigeria, and South Africa.^25^ As discussed in that parent study on all-cause mortality, the Community Knowledge Approach (CKA) method harnesses the collective knowledge of a given community to identify deaths, usually involving both lay individuals and key informants such as community leaders and health workers.^26^ It was employed to overcome some of the limitations and challenges of existing mortality measurement among FSW populations, including: the absence or incompleteness of civil registry or vital statistics systems; poor health service utilisation and high numbers of out-of-facility deaths in this group, limiting scope for using hospital reports; the logistical challenges (and associated time and expense requirements) of conducting surveys among a highly mobile population with distrust for authorities, thereby limiting application of maternal mortality estimation techniques such as the Sisterhood method; and the limitations of verbal autopsies convened with family members who often live far away from where the FSW worked and who may have limited knowledge of FSW employment and/or the circumstances of the death.^2,27,28^ In contrast, the research team’s previous experience working with FSW communities demonstrated that FSW peers are very knowledgeable about how other FSW in their community die and are willing to discuss the details of the deaths within the context of a community group.^22^

In a community study in Bangladesh, the CKA method was previously found to have high sensitivity in the identification of maternal deaths (100%) and neonatal deaths (80%), when validated against reporting within household surveys.^29^ It builds on several other community informant methodologies which have been successfully used to identify maternal deaths.^30–32^ Given the existent social networks of FSW who constituted a coherent “community”, it was therefore considered an ideal and cost-effective method to understand the causes and circumstances of deaths within this vulnerable population.

The Bangladesh CKA was modified for this study: by convening FSW to a group meeting, to represent the wider FSW community network (in Bangladesh community residents were identified and interviewed at village gathering points (tea stalls, local markets)); and by relying on informant data alone (in Bangladesh deaths were confirmed by additional key informants and cause of death was confirmed with the household).

The contexts for the eight study countries are summarised in Table 1. Abortion rates are highest in India, followed by DRC and Kenya. Maternal mortality is highest in Nigeria. Three countries have highly restricted access to abortion (Angola, Indonesia, and Nigeria); three are moderately restricted, with abortion allowed under certain circumstances (Brazil, DRC, and Kenya); and two are liberal, with abortion theoretically freely accessible through the public sector (India, South Africa), although in practice these are contexts where unsafe abortions are still frequently procured.^1, 2^ Abortion incidence is high across all the study countries, ranging from 25 to 48 per year per 1000 women of reproductive age.^4^ According to IHME and its associated Global Burden of Disease study, abortion deaths comprise the largest fraction of maternal deaths in India (11.4%) and Kenya (10.5%).^33^

**Table 1.** Summary of abortion and maternal health status across the eight study countries

	Unintended pregnancy rate (2015–2019, per 1000 women aged 15–49)^34^	Abortion rate (2015–2019, per 1000 women aged 15–49)^34^	Maternal mortality rate, per 100,000 live births (2017)^35,36^	Annual nos. maternal deaths (2017, IHME)^33^	Annual nos. deaths from abortion* (% of all maternal) (2017, IHME) ^33^	Legal restrictions around abortion^37^	De facto access to abortion
Angola	120	33	241	2126	198 (9.3%)	Highly restricted: legal only to save a woman’s life.	PAC (surgical or medical) only. Limited distribution of misoprostal.^38,39^
Brazil	67	32	60	2054	206 (10.0%)	Moderate: In cases of rape, to save a woman’s life, and in the cases of anencephaly.	Abortion is common, but many are obtained unsafely.^17^
DRC	117	44	473	10,166	1025 (10.1%)	Moderate: To save a woman’s life and to preserve her health, with explicit mention of mental health	Safe services are largely inaccessible despite new legal framework. Limited social marketing and private sales of MA in urban areas.^40^
India	62	48	145	39,427	4478 (11.4%)	Liberal: Permitted under a broad range of socio-economic circumstances (including rape, contraceptive failure, fetal impairment)	Limited number of facilities offering abortion. MA commonly available over the counter, accessed with and without prescription.^41^
Indonesia	40	25	177	6627	656 (9.9%)	Highly restricted: in medical emergencies, and in cases of severe fetal anomaly	The vast majority of abortions occur outside these legal parameters and many occur under unsafe conditions.^42^
Kenya	113	43	342	3990	418 (10.5%)	Moderate: To save a woman’s life and to preserve her health (but with no explicit mention of mental health)	Safe services remain restricted to a small number of hospitals and private clinics. Limited social marketing and private sales of MA.^43^
Nigeria	68	33	917	17,982	1062 (5.9%)	Highly restricted: To save woman’s life.	Women mostly self-manage abortion with non-recommended methods outside the formal healthcare system.^44^
South Africa	81	30	119	1191	88 (7.4%)	Liberal: No restrictions up to 12 weeks. In second trimester, allowed under certain circumstances (health risk, rape/incest, socio-economic impact).	Freely available in the public sector, but geography and provider attitudes restrict access; high proportion sought in the informal sector.^10^

Note: MA = medical abortion.

*Includes spontaneous & induced abortion and deaths from ectopic pregnancy.

In the parent study, abortion was identified as the leading cause of death by the 1280 FSW who participated in the broader study and who reported on FSW deaths within their social network.^25^ This current analysis further examines the abortion-related mortality in this population and aims to understand in detail how these deaths occurred, including place of death, method of abortion used, and gestational age at death. To our knowledge, this is the first study investigating the details of FSW abortion-related mortality, including abortion methods used.

## Methodology

### Design and participants

Study methods were described in detail in the first paper reporting on all-cause mortality.^25^ In brief, we used the CKA to identify deaths and their causes within primarily urban FSW social networks in the eight countries, which was part of a broader investigation of the maternal and child health of this population.

FSW were recruited through local sex worker organisations and non-governmental organisation (NGO) networks in urban areas (1–7 cities per country). The NGOs were partners known to the lead researcher or recommended by other FSW researchers. Potential FSW participants were approached in bars, brothels, and open spaces (street, parks, fields) and invited to participate in a group meeting to report on the health and deaths of peer FSW in their social networks. The research team and partner NGOs aimed to have 10–12 participants attend per group, to allow time for reporting. Refusal rates were not recorded. The group format was selected as a means to engage the “community” of the CKA methodology, thus minimising duplicate reporting and increasing efficiency of research staff time. Potential participants were screened on these criteria: (1) aged at least 18 years; (2) mother to at least one child aged 10 or younger (due to broader study questions on child health); (3) engaged in full-time sex work for at least three years prior to the study; and (4) interactive with other FSW in the community. Following an informed consent process, group meetings were conducted in locations that provided participants with privacy during the group discussion. They were facilitated by an English-speaking lead researcher. Discussions in Angola, Brazil, DRC, India, and Indonesia were co-facilitated by NGO staff (in the local language) and the lead researcher.

### Data collection and quality assurance

In total 165 group meetings were convened between January and October 2019 across 24 cities* in the eight countries (12–32 groups per country; 2–15 groups per city). Within cities, each group was held in a different location (ward/neighbourhood) in order to capture local community deaths. During the meeting, participants were asked to report on the deaths of any FSW in their social network since the beginning of 2014, as well as the causes of these deaths. Participants took turns to report on deaths they knew about, which also helped reduce double-counting. In the rare instances where deaths were known about by more than one participant, and/or where the cause of death was complicated (e.g. involving both pregnancy and violence), a short discussion ensued to determine the ultimate cause of death. Indicators gathered in the structured questionnaire included first name of deceased (or pseudonym), age at death, number of children, year of death, city where death occurred, location where the death occurred (home, hospital, informal clinic, other), pregnancy status, whether the death followed an abortion (and if so, the type of abortion, i.e. surgical, medical, or other), whether the death occurred during childbirth or immediately postpartum, the reported cause of death (as abortion, murder, HIV/AIDS, suicide, accidents, and other causes). Extensive hand-written notes, including verbatim quotes, were also recorded by the lead researcher. Following each group meeting, the lead researcher and local partner together reviewed all deaths reported across all discussion groups in that location and identified potential duplicates. Additionally, during data cleaning, all deaths across a city were reviewed and any further duplicates identified were removed. If any two deaths matched on two reported details, only the details of the first death were recorded. Local partners also ensured that no FSW participated in more than one group.

### Measures

Maternal deaths were coded in accordance with the ICD-MM classification by the lead researcher and an obstetrician/gynecologist.^45^ Since only year of death was recorded, not month, annual numbers of abortion-related deaths were calculated by adding together deaths for 2018 and 2019, and annualising the total based on the date (month) of the group discussion in 2019. Method of abortion causing the death was categorised as use of: traditional medicine (herbs, local medicine), detergent/bleach, probes/needles or other invasive procedure, unknown medication (pills, injection), salt and coke/water, surgical abortion procedure (including surgical PAC) or via an unknown method. Gestational age was reported in months.

### Data entry and analysis

Data were entered into an Excel database, and cleaned by three researchers for accuracy, completeness, and duplication using the original records. Descriptive statistics are used to report the number of deaths, causes of deaths, death by type of abortion, location of death, gestational age at time of death, and number of children left motherless, by country. Since the CKA was used to capture *all* deaths known by participants, no statistical comparisons between countries are made. Differences between countries should still be interpreted with caution, however, given the small number of abortion deaths in some countries, and the non-systematic sampling of FSW communities of participants.

### Ethics

The study protocol, consent forms, and questionnaire were reviewed and approved by the Institutional Ethics Review Board of Portland State University, USA (Protocol #184888, 3rd January 2019). While obtaining national ethics approval in each country would have been ideal, this was not required by the US board; instead the research team asked each local partner to review the forms for local standards and approve their use.

### Results

Altogether, 1280 FSW participated in the group discussions, who reported a total of 2112 unique FSW deaths from within their social network (ranging from median of 0.49 per participant in Brazil, to 3.02 per participant in Kenya) (Table 2).

**Table 2.** Mortality among FSW, by country

	No. of group participants	Total no. FSW deaths reported	Mean no. deaths reported per FSW participant	No. (%) deaths due to maternal causes (*n* = 2112)	No. (%) maternal deaths due to unsafe abortion (*n* = 1320)	Annual unsafe abortion death count (2018/2019)
Angola	71	52	0.73	6 (11.5)	4 (66.7)	3
Brazil	80	39	0.49	1 (2.6)	1 (100.0)	1
DRC	270	606	2.24	537 (88.6)	304 (56.6)	177
India	152	132	0.87	43 (32.6)	19 (44.2)	4
Indonesia	76	58	0.76	12 (20.7)	4 (33.3)	2
Kenya	175	529	3.02	312 (59.0)	188 (60.3)	116
Nigeria	312	527	1.69	386 (73.2)	216 (60.0)	130
South Africa	144	169	1.17	23 (13.6)	14 (60.9)	4
Total	**1280**	**2112**	**1**.**65**	**1320 (62.5)**	**750** (**56.8)**	**437**

In total, 1320 (62.5%) deaths were due to maternal causes, ranging from 2.6% of all FSW deaths in Brazil to 88.6% in DRC. Among the maternal deaths, 750 (56.8%) were due to unsafe abortion. Unsafe abortion comprised over half of the maternal deaths reported in Angola, Brazil, DRC, Kenya, Nigeria, and South Africa. Participants in DRC recorded the highest number of deaths from unsafe abortion (304, reported by 270 participants), followed by Kenya (188 deaths reported by 175 participants), and Nigeria (216 reported by 312 participants). In total, 437 abortion-related deaths occurred in the most recent one-year period, with annual numbers highest in DRC (177), Nigeria (130), and Kenya (116). Other causes of all deaths and of maternal death are reported in Willis et al.^25^

Table 3 presents detail on the deaths caused by unsafe abortion (*n* = 750). The mean age at death was 24.6, ranging from 23.0 in DRC and South Africa to 33.5 in Angola. Mean gestational age at time of death was 4.6 months (in the sample with known duration of pregnancy (*n* = 615; 82.0%)). In DRC and Nigeria, an important proportion of deaths was occurring in the third trimester (months 7–9) (*n* = 49 (17.1%) and *n* = 36 (17.5%) respectively) (data not shown). Three-quarters of the abortion deaths occurred outside of hospital, either at home or elsewhere. Most deaths (73.9%) were among women with children, and these deaths left 1,207 motherless children, with the greatest number in DRC (*n* = 524).

**Table 3.** Deaths from unsafe abortion, by country

Country (no. of abortion deaths)	Mean age at time of death from abortion	Gestational age (months) at time of death (mean (SD) (N))*	% of abortion deaths outside hospital (home or other) (N)*	% abortion deaths resulting in motherless children	No. motherless children following abortion deaths
Angola (*n* = 4)	33.5	Unknown	0 (1)	100	12
Brazil (*n* = 1)	Unknown	Unknown	0 (0)	0	0
DRC (*n* = 304)	23.0	4.6 (1.9) (286)	66 (301)	75.0	524
India (*n* = 19)	27.4	3.8 (1.2) (6)	100 (3)	58.0	17
Indonesia (*n* = 4)	26.8	Unknown	0 (0)	25.0	2
Kenya (*n* = 188)	25.6	4.8 (1.7) (116)	70 (151)	74.5	263
Nigeria (*n* = 216)	25.5	4.6 (1.7) (206)	91 (201)	74.5	376
South Africa (*n* = 14)	24.6	7.0 (–) (1)	0 (0)	64.3	13
Total (*n* = 750)	24.6	4.6 (1.8) (615)	75 (658)	73.9	1207

*Excludes missing data, denominator N with data shown in brackets.

Table 4 presents data on the methods of abortion resulting in death. The highest number of unsafe abortion deaths (37.9%) followed use of traditional medicines (such as herbs or local medicines). Consumption of unknown or unsafe pills, injections, or medicines was also common (29.9%). Other less frequently used methods were surgical procedures; use of probes, needles or other invasive instruments; consumption/insertion of detergent/bleach, or salt and coke/water. Methods of abortion resulting in death varied by country context: consumption of unknown medications was most common in DRC (38.5%); whereas traditional medicines were most common in Nigeria (52.3%) and Kenya (30.3%). Participants from DRC were the only ones to report the use of salt and coke/water.

**Table 4.** Methods of unsafe abortion resulting in death*

Country (n)	Traditional medicines (herbs, local medicine) (*n* (%))	Detergent, bleach (ingested or inserted vaginally) (*n* (%))	Probes, needles or other invasive procedure (*n* (%))	Unknown medication (pills, injection) (*n* (%))	Salt and coke/water (*n* (%))	Surgical procedure (*n* (%))	Unknown method or other (*n* (%))	Total (*n* (%))
Angola (*n* = 4)	0 (0%)	0 (0%)	0 (0%)	0 (0%)	0 (0%)	0 (0%)	4 (100%)	4 (100%)
Brazil (*n* = 1)	0 (0%)	0 (0%)	0 (0%)	1 (100%)	0 (0%)	0 (0%)	0(0%)	1 (100%)
DRC (*n* = 304)	114 (37.5%)	3 (1.%)	0 (0%)	117 (38.5%)	20 (6.6%)	17 (5.6%)	33 (10.9%)	304 (100%)
India (*n* = 19)	0 (0%)	0 (0%)	0 (0%)	9 (47.4%)	0(0%)	8 (42.1%)	2 (10.5%)	19 (100%)
Indonesia (*n* = 4)	0 (0%)	0 (0%)	0 (0%)	0 (0%)	0 (0%)	0 (0%)	4 (100%)	4 (100%)
Kenya (*n* = 188)	57 (30.3%)	6 (3.2%)	8 (4.3%)	48 (25.5%)	0 (0%)	22 (11.7%)	47 (25%)	188 (100%)
Nigeria (*n* = 216)	113 (52.3%)	0(0%)	0 (0%)	48 (22.2%)	0 (0%)	47 (21.8%)	8 (3.7%)	216 (100%)
South Africa (*n* = 14)	0 (0%)	0 (0%)	0 (0%)	1 (7.1%)	0 (0%)	1 (7.1%)	12 (85.7%)	14 (100%)
Total (*n* = 750)	284 (37.9%)	9 (1.2%)	8 (1.1%)	224 (29.9%)	20 (2.7%)	95 (12.7%)	110 (14.7%)	750 (100%)

*Some women discussed use of multiple methods and abortion attempts. The final method resulting in death is recorded here.

## Discussion

This study has identified large-scale preventable maternal mortality from unsafe abortion occurring among FSW across a range of LMICs. The 750 maternal deaths due to unsafe abortion reported in the peer networks of the 1280 research participants demonstrate unequivocally the frequency with which the lives of vulnerable and marginalised women are placed at risk by the lack of access to safe abortion and PAC services.

The highest numbers of abortion-related deaths were recorded by FSW participants in DRC, Kenya and Nigeria, all settings that suffer from poor access to safe abortion care, through a combination of legal restrictions on abortion provision and/or poor availability of and access to safe services. Marginalised populations in these settings may experience particularly poor access to safe abortion care, as has been previously demonstrated in a study among Kenyan urban slum-dwellers.^43^ Preventable deaths from unsafe abortion were also occurring in the more liberal policy contexts of South Africa and India, demonstrating the persistence of barriers to safe legal care within the formal health system for these populations. This has been shown in other studies which document continuing recourse to unsafe methods there.^10, 41^ While previous research has demonstrated the scale of unintended pregnancy and practices of unsafe abortion in the eight study contexts, to our knowledge this is the first study to shine a light on the vast scale of preventable mortality being experienced by this particular population.

The extremely high numbers of deaths from abortion recorded in this study are very unlikely to be explained by physiological causes alone. For example, there is no evidence that abortion complications are impacted by HIV status.^46^ Instead, the current study clearly demonstrates the enduring recourse to highly unsafe methods of abortion. FSW who died from abortion complications in this study most frequently relied on traditional medicine and herbs and illicit and unregulated medication. A small but notable proportion also died following a surgical procedure: extensive notes taken during the surveys demonstrated that these were almost exclusively provided by informal and unregulated providers, often referred to as “quacks” by participants.

It is challenging to contrast this study’s results with existing research, since little robust data on deaths from unsafe abortion in these contexts exists, and there is no literature on abortion deaths among FSW. Many studies on causes of maternal death are based on hospital records that fail to capture deaths from unsafe abortion occurring in the community. In Nigeria, for example, hospital-based studies find unsafe abortion causes less than 10% of maternal deaths, which is a fraction of the 60% identified in this study’s FSW population.^47,48^ Even WHO, which has attempted to include community deaths in its estimations, still only finds unsafe abortion as causing 10% of maternal deaths in the sub-Saharan region.^2^ Studies conducted among marginalised communities find more elevated estimates. A study in Nairobi’s urban slums found very high maternal mortality, with 31% attributed to abortion complications.^49^ This is still only half of the 60% found among Kenyan FSW in this study, however.

Comparisons of the numbers of FSW deaths with national estimates can be more illuminating. Table 5 presents an indication of how the reported numbers of FSW deaths in this study compare with national abortion-related death estimates for 2017 reported by IHME, which also provide estimates of the numbers of abortion deaths (unlike other global estimates).^33^ These comparisons must be interpreted with caution because IHME’s estimates are lower than UN estimates in some countries;^2,50^ and because the annual FSW deaths from this study are approximated based on both 2018 and 2019 data. It’s also likely that many of the FSW deaths are not currently included within national estimates (given so many occurred outside of hospital settings) and so presenting the study deaths as a proportion of all national deaths may overestimate their contribution to total mortality. Notwithstanding these caveats, the table shows that the deaths reported by the 1280 FSW participants constituted an important proportion of the total maternal deaths caused by abortion, even within these limited geographic settings. Notably, in Kenya, the abortion-related annualised deaths reported by this sample of 175 FSW comprise over a quarter of abortion deaths nationally. In DRC and Nigeria they also comprise an important fraction of all national abortion deaths, at 17% and 12%, respectively. It is therefore imperative to ensure that FSW deaths are robustly measured in national maternal death monitoring.

**Table 5.** Comparison of study estimates to nationally representative data

	No. participants	Annualised abortion death total*	IHME 2017 abortion deaths†	Study FSW % of national abortion deaths
Angola	71	3	198.0	1.5
Brazil	80	1	205.6	0.5
DRC	270	177	1024.8	17.3
India	152	4	4477.5	0.1
Indonesia	76	2	656.1	0.4
Kenya	175	116	417.9	27.7
Nigeria	312	130	1062.4	12.3
South Africa	144	4	87.9	4.0

*Annualised from 2018 + 2019 deaths, according to the month of data collection in 2019 (Jan–Sept) as reported previously^22^

†IHME estimates for abortion deaths include spontaneous abortion.

The late average gestational age at time of death, commonly during the second trimester, is likely to be an important risk factor for the high mortality experienced in this population. It notable that around one in six of the deaths in Nigeria and DRC were even occurring in the third trimester. Studies from PAC settings in Kenya and Nigeria indicate that late abortions requiring surgical intervention are common, with more severe complications occurring among women presenting at later gestational ages.^51,52^ Severe abortion complications are also higher in the second trimester among women who interfere with the pregnancy themselves.^43^ Later-term legal abortions (over 12 weeks) are severely restricted in all the countries studied, except South Africa and India, which likely contributed to the unsafe method choices made by the deceased.^37^

Existing studies point to a range of systemic, legal, structural, and socio-economic barriers that hinder access to safe abortion and PAC for all women in LMIC settings, even where some abortion care is theoretically available through the public health system.^1,53^ Further operational research among FSW is needed to understand the specific barriers they face – is it a question of knowledge, cost, supply, stigma, feared criminality, or other unknown factor inhibiting access to safer abortion products and care? Notes taken during the group meetings suggested that costs and stigma were two key determinant factors, in particular when emergency PAC may have saved the woman’s life. This is supported by other studies that demonstrate the challenges of cost and stigma that FSW face when trying to access formal health services.^3^ The late mean gestational age at time of death also suggests that timely diagnosis of pregnancy may be problematic in this group. The diffusion of harmful abortion practices may also be exacerbated by the exclusion of FSW social networks from mainstream society and information sources.^54^ But these networks also offer opportunities for the diffusion of public health information, for example on the availability of contraception, and ways to access safe medical abortion products (misoprostol alone or in combination with mifepristone). And while not measured explicitly in this study, high numbers of deaths from abortion indicate high levels of unintended pregnancy in this group: further investigation into poor access to and use of effective contraception among FSW is warranted.

Further research on the causes of unsafe abortion should, however, be integrated with action to mitigate the problem. International health agencies have recommended expanding the scope of SRH care provided to FSW: the 2013 *Implementing Comprehensive HIV/ STI Programmes with Sex Workers: Practical Approaches from Collaborative Interventions,* produced by six international agencies including UNAIDS and WHO, explicitly recommends the provision of abortion care to this population.^13^ STI and HIV programmes that have had success in reaching this unstable and transient population also need to ensure delivery of quality contraceptive services, including more effective longer-acting methods such as injectables, implants, and intra-uterine devices, in addition to condoms for STI/HIV prevention. Family planning programmes need to ensure outreach to FSW communities. Where programmes are prevented from referring for abortion care (as is the case with all US-funded HIV programmes), mechanisms must be identified to inform FSW of other organisations offering or providing information on safe abortion care and PAC. Misoprostol, commonly accessible for the treatment of postpartum haemorrhage or gastric ulcers, has been shown to substantially reduce the toll of abortion-related mortality,^5^ and is available for distribution and use in all eight countries included in this study, including the more restricted settings of DRC and Nigeria.^39^ Educating FSW on its use could be impactful in reducing deaths. Abortion accompaniment models may be particularly beneficial for this group, since they have been shown to improve the safety of and access to supported safe abortion in legally-restricted settings.^55^

It is important to note some methodological strengths and limitations of the study. Capturing reports on abortion experiences and outcomes is always challenging due to the stigmatised nature of the procedure and the legal and policy contexts in which they occur.^2^ Recording abortion-related deaths among a marginalised and frequently criminalised population is even more challenging. The use of the CKA and its group discussions created a safe environment in which participants felt unthreatened by their reporting of potentially illicit abortions among their deceased peers. While the methodology was not able to generate precise death rates or accurate comparisons between countries, principally because reporting timeframes varied and more distal deaths may not have been well-documented, the high frequency and detail with which FSW reported deaths in their peer networks give confidence in the veracity and scale of the reports. Many of these deaths would have been missed by hospital records, given that so many occurred outside the formal health system setting, and the CKA also overcame the limitations of verbal autopsy reports which are commonly given by family members – many of whom may be unaware of or unwilling to admit the deceased’s sex work, and likely had little knowledge of the details surrounding the deaths occurring far away from the family home. It is also very likely that the stigma surrounding abortion and/or pregnancy out-of-wedlock also inhibits discussion of abortion-related deaths in a verbal autopsy. Rather, the peer FSW in this study were able to report the details of the deaths of other FSW in their social network following unsafe abortion, facts that would have been unavailable to most family members.

It is possible that some of the reported abortions could have been “safe” procedures (according to WHO, either performed by trained providers using a recommended methods, or using a safe method (e.g. misoprostol) with adequate support),^1^ and were misclassified here as unsafe. This is unlikely, however, given the demonstrated safety of regulated surgical abortion and PAC in low-income settings,^53^ and subsequently very low risk of death. Conversely, the consequences of unsafe abortion may have been even greater than the deaths reported in this study. It is likely that the total mortality burden in the study’s reporting period (5–6 years) was underestimated: 82.5% of the deaths reported occurred after January 2018 (12–18 months preceding the survey), indicating that respondents may have suffered from recall bias in reporting of more distal deaths. In Angola, Brazil, India, and Indonesia, data collection periods and locations were limited by logistical challenges. In Kenya, the team ran out of time to record all the known deaths since there were many more than anticipated. Thus the total number of deaths reported in these settings is lower than might otherwise have been the case. Using mothers as informants may also have under-estimated deaths among younger FSW. The total number of deaths in each study location may also have been under-estimated since only select NGOs were used to identify FSW, and not all FSW known to the NGOs and who met the inclusion criteria participated in the groups.

Another limitation of our study is a lack of complete data on the abortion history and context leading up to the death. Research from legally restricted settings demonstrates how women often use multiple illicit methods to try to terminate a pregnancy.^56^ The complexity of these methods and timeline of different abortion attempts were not possible to record with the research methods used. The fact that most abortions were occurring in the second trimester suggests that women were either unaware of their pregnancy, were unable or unwilling to make a decision to terminate the pregnancy, or had made previous unsuccessful attempts to do so. It is also possible that the causes of death may have been misreported or misclassified. On the one hand, there was potential visibility bias, with some deaths from unsafe abortion potentially being unknown, for example, if the death occurred during the first trimester before others knew of the pregnancy. But conversely, the very high proportion of all deaths due to unsafe abortion was surprising, and results contrast starkly with previous estimates on causes of maternal deaths in all study countries. Questions used to record all-cause mortality and the details of the death were not previously validated, and there was some scope for misclassification by the research team. Furthermore, in contrast to the original Bangladesh CKA, it was not possible to verify the cause of death with a third party. There is no apparent reason, however, to mistrust the reporting by the FSW participants. Rather, the use of the CKA allowed open and frank discussion about the circumstances of the deaths of peers.

The study findings point to a potential research agenda on the reproductive health of FSW. A deeper understanding of the lived reproductive experiences of FSW could elucidate how the social and economic context of their marginalised lives influences their sexual practices, contraceptive use, patterns of menstruation, pregnancy diagnosis, and abortion-seeking behaviour. Research could also examine how their vulnerability to control, exploitation, and physical violence impact on decision-making and the achievement of reproductive autonomy. Further research should also measure mortality *rates* in this population, to allow for accurate comparison with other population groups. This may require methodological innovation, to measure both the population size of FSW as well as the deaths occurring within this group, and/or the implementation of community death registration systems. The latter may prove challenging, however, as long as sex work remains criminalised. More evidence is also needed on unsafe abortion-related morbidity in this population, including impacts on disability, lost employment, economic hardship, and mental health. Generating this body of evidence can support the design of an effective programmatic response to address the severe mortality being experienced by some of the world’s most vulnerable women. It can also help provoke the urgently needed international and domestic attention and financing required to support comprehensive SRH programmes.

This study has demonstrated that unsafe abortion is an important cause of death among many of the FSW communities included in this study. Decisive action from governments, funders, and NGOs, is now needed to provide essential SRH care to this marginalised group, including vulnerable women presently in sex work and those who may enter sex work. Current and looming structural threats – including the COVID-19 pandemic, food insecurity, inflation, conflicts, and climate change – are only likely to increase the vulnerability of FSW, and without concerted action, the consequences will be many more preventable deaths.

## Author contributions

BW was responsible for study conceptualisation, research methodology, funding acquisition, project administration, and data collection and curation. KC was responsible for analysis, writing the original draft, and reviewing and editing the manuscript. EP was responsible for data curation, and analysis. HT assisted with data curation and analysis. SW supported data curation and analysis. WMK assisted with investigation and data analysis. All authors reviewed and edited the manuscript. All authors had full access to study data and take responsibility for the final publication submission.

## Footnotes

* Cities (no. of groups): Abuja (10), Bangalore (3), Bukavu (15), Calabar (7), Cape Town (4), Chennai (2), Durban (5), Gudibanda (2), Hyderabad (2), Jakarta (13), Johannesburg (7), Kinshasa (12), Kisumu (10), Lagos (15), Luanda (12), Mombasa (14), Nairobi (12), Nashik (4), Port Shepstone (2), Rio de Janeiro (5), Salem (4), Salvador (2), Sau Paulo (8), Warangal (2).

## Data sharing

De-identified aggregate data used for this analysis can be requested from the corresponding author after publication. No other documents will be made available. Access permission will be considered based on the following criteria: (a) request should be for the purpose of partnering on research on female sex workers; or (b) for inclusion in curriculum for educational purposes; or (c) for providing services to female sex workers by governmental organizations, SWOs, and NGOs. Request for one of these purposes will be considered from governmental organizations, SWOs, or NGOs providing support to FSW, or researchers from recognised institutions.
